# Integration of Gender-Affirming Primary Care and Peer Navigation With HIV Prevention and Treatment Services to Improve the Health of Transgender Women: Protocol for a Prospective Longitudinal Cohort Study

**DOI:** 10.2196/14091

**Published:** 2019-06-27

**Authors:** Javier R Lama, Kenneth H Mayer, Amaya G Perez-Brumer, Leyla Huerta, Hugo Sanchez, Jesse L Clark, Jorge Sanchez, Sari L Reisner

**Affiliations:** 1 Asociacion Civil Impacta Salud y Educacion Lima Peru; 2 The Fenway Institute Boston, MA United States; 3 Department of Sociomedical Sciences Mailman School of Public Health Columbia University New York, NY United States; 4 Epicentro Lima Peru; 5 Department of Medicine Geffen School of Medicine University of California Los Angeles Los Angeles, CA United States; 6 Centro de Investigaciones Tecnologicas, Biomedicas y Medioambientales Universidad Nacional Mayor de San Marcos Lima Peru; 7 Division of General Pediatrics Boston Children’s Hospital Harvard Medical School Boston, MA United States; 8 Department of Epidemiology Harvard TH Chan School of Public Health Boston, MA United States

**Keywords:** transgender persons, culturally competent care, patient navigation, HIV, retention in care, health services, Peru

## Abstract

**Background:**

Public health strategies are urgently needed to improve HIV disparities among transgender women, including holistic intervention approaches that address those health needs prioritized by the community. Hormone therapy is the primary method by which many transgender women medically achieve gender affirmation. Peer navigation has been shown to be effective to engage and retain underserved populations living with HIV in stable primary medical care.

**Objective:**

This study aims to assess the feasibility and acceptability of an integrated innovative HIV service delivery model designed to improve HIV prevention and care by combining gender-affirming primary care and peer navigation with HIV prevention and treatment services.

**Methods:**

A 12-month, nonrandomized, single-arm cohort study was implemented in Lima, Peru, among adult individuals, assigned a male sex at birth, who identified themselves as transgender women, regardless of initiation or completion of medical gender affirmation, and who were unaware of their HIV serostatus or were living with HIV but not engaged in HIV treatment. HIV-negative participants received quarterly HIV testing and were offered to initiate pre-exposure prophylaxis. HIV-positive participants were offered to initiate antiretroviral treatment and underwent quarterly plasma HIV-1 RNA and peripheral CD4+ lymphocyte cell count monitoring. All participants received feminizing hormone therapy and adherence counseling and education on their use. Peer health navigation facilitated retention in care by visiting participants at home, work, or socialization venues, or by contacting them by social media and phone.

**Results:**

Patient recruitment started in October 2016 and finished in March 2017. The cohort ended follow-up on March 2018. Data analysis is currently underway.

**Conclusions:**

Innovative and culturally sensitive strategies to improve access to HIV prevention and treatment services for transgender women are vital to curb the burden of HIV epidemic for this key population. Findings of this intervention will inform future policies and research, including evaluation of its efficacy in a randomized controlled trial.

**Trial Registration:**

ClinicalTrials.gov NCT03757117; https://clinicaltrials.gov/ct2/show/NCT03757117

**International Registered Report Identifier (IRRID):**

DERR1-10.2196/14091

## Introduction

Globally, transgender women (TW) are at a high risk for HIV infection, with a pooled 19.1% prevalence and a 48.8-fold increased odds of HIV compared with the general adult population [1]. Public health strategies are urgently needed to improve global HIV disparities among TW, including intervention approaches that address those health needs prioritized by the community [2]. In Peru, TW are disproportionately burdened by the HIV epidemic, with HIV prevalence estimates ranging from 30% to close to 45% [3-5] compared with less than 1% in the general population [6]. About 22,500 TW live in Lima (personal communication by Segura et al, 2010), the national capital and regional center, which is home to 80% of Peru's HIV epidemic [7], making it a key site for HIV interventions in Latin America. Improving access to HIV prevention and treatment services for TW in Peru is vital to curb the burden of HIV epidemic for this key population [8].

Gender affirmation is defined as the process of being recognized in one’s internal felt gender and sense of oneself as having a particular gender identity [9,10]. Access to gender affirmation has been conceptualized across multiple domains: social (eg, *passing* in one’s desired gender role, acceptance and use of preferred pronouns or name, and wearing desired clothes associated with gender identity), medical (eg, hormone therapy or surgery), and legal (eg, legal name change, change of birth certificate sex or passport/ID reflecting gender identity) [11]. Not all types of affirmation are needed or desired by transgender individuals. Hormone therapy is the primary method by which many TW medically achieve gender affirmation [12], as some consider body modification an important step in aligning their outward physical gender presentation with their internal felt sense of their gender [10]. The implementation of gender-affirming care has been shown to improve psychological well-being among TW [11,13]. Furthermore, studies suggest that the integration of gender-affirmative care, including delivery of feminizing hormone therapy, may facilitate TW engagement in HIV care [14,15]. Integrating hormone therapy with HIV prevention and care may increase patient-provider trust, provide an opportunity for patients and providers to discuss what is known about drug-drug interactions between antiretroviral medications and hormones shown to impede HIV prevention and treatment uptake and adherence among TW, foster positive interactions, reduce barriers to obtaining needed services, and support ongoing engagement and retention in HIV care [15,16].

Further evidence from Peru highlights lack of medical training and insufficient culturally competent clinical services to implement feminizing hormone therapy for TW [17]. As a result, gender affirmation procedures are usually performed outside the health system in informal peer-delivered systems [4]. In this setting, trained TW community members, as educators and/or peer navigators, could improve gender-affirming medical care and also be involved into and support HIV service delivery programs, as research demonstrated the effectiveness of peer navigation to engage and retain underserved populations living with HIV in stable primary medical care [18].

In recent years, the concept of an HIV treatment cascade has emerged as a way to identify gaps in the continuum of how well people living with HIV are engaged in medical care. It consists of 5 main steps, including diagnosis, linkage to care, retention in care, adherence to antiretroviral therapy, and viral suppression [19]. Similarly, an HIV prevention cascade model has been proposed to assist in the implementation and monitoring of HIV prevention programs by identifying gaps in the steps required for effective use of prevention methods, including motivation, access, and effective use in a priority population that would benefit from the prevention method [20].

The *Féminas* (the plural form of *Fémina* in Spanish, meaning *Feminine* in English) study was designed to assess the feasibility and acceptability of a service delivery model designed to improve the HIV treatment and prevention cascades among TW in Lima, Peru, by integrating HIV prevention and care services with gender-affirming transgender medical care supported by peer navigation. The study was grounded in an implementation science framework, which aimed to test and translate research to promote evidence-based practices for improving health and well-being [21,22]. Specific design considerations were given to elements that could be replicated by different institutions, including the Ministry of Health, nongovernmental organizations and community-based organizations (CBOs) providing HIV prevention and care for TW, and/or researchers seeking to develop health interventions culturally tailored to the needs of TW. A mixed-methods formative research study was conducted to explore barriers and facilitators to implementing the proposed model of care. Perceived acceptability of the integrated care model was high among TW (n=48) and health care professionals (n=19) alike. Barriers for implementation included stigma, lack of provider training or Peruvian guidelines regarding optimal TW care, and service delivery obstacles (eg, legal documents, spatial placement of clinics, and hours of operation). The hiring of TW staff was identified as a key facilitator for engagement in health care [17,23]. These findings informed that working in partnership with local TW and health care provider organizations is critical to overcoming existing barriers to successful implementation of an integrated HIV prevention and treatment services and gender-affirmative medical care model for TW in Peru.

## Methods

### Study Design

**Figure 1:**
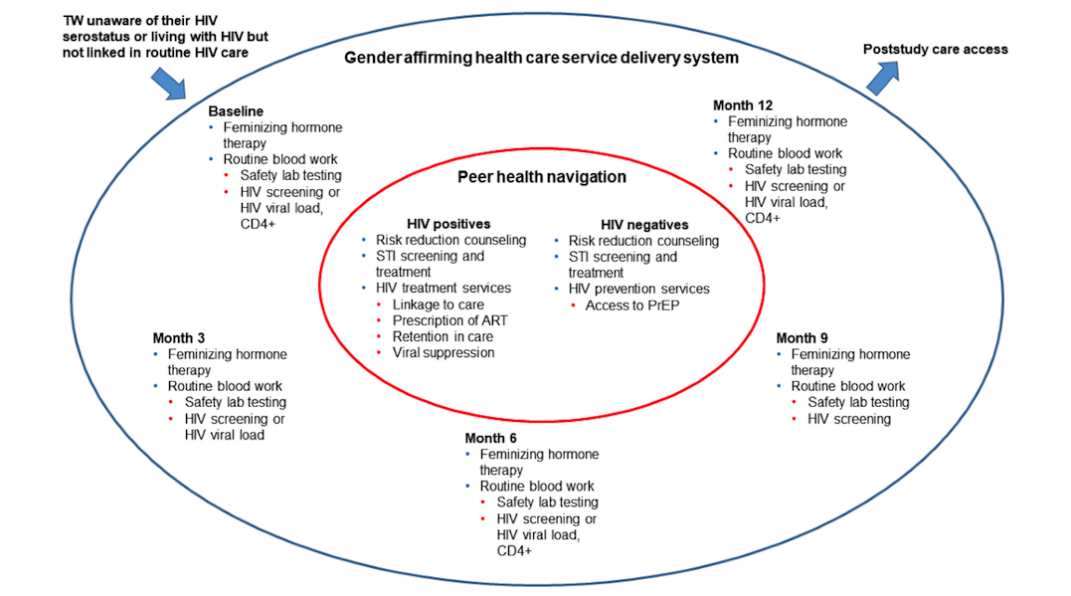
Schematic of gender-affirming health care and peer navigation to improve the HIV prevention and treatment cascades. ART: antiretroviral therapy; PrEP: pre-exposure prophylaxis; STI: sexually transmitted infection; TW transgender women.

Between October 1, 2016 and March 31, 2017, a 12-month, nonrandomized, single-arm cohort study was enrolled in Lima, Peru, to assess the feasibility and acceptability of integrating routine HIV prevention and treatment services with gender-affirming care (ie, feminizing hormone therapy) supported by TW community peer health navigators (Figure 1).

The study was led by Asociacion Civil Impacta Salud y Educacion (IMPACTA), a nongovernmental HIV research organization, in partnership with Epicentro, a CBO that provides low-cost HIV and sexually transmitted infection (STI) testing and care, community services, and hosts volunteer-driven community activities, which served as a research site for all study operations. The Fenway Institute at Fenway Health, a leading center of transgender clinical care, training, education, and research in Boston, Massachusetts, United States, served as a research partner. Fenway Health’s informed consent model of transgender care and community-based transgender health research [24] were the basis for the *Féminas* intervention in Lima, Peru.

### Entry Criteria

Adult individuals, aged 18 years or older, assigned a male sex at birth, who identified themselves as TW or on the trans-feminine continuum (eg, *trans*, *travesti*, *transgender*, or *transsexual*), regardless of initiation or completion of medical gender affirmation procedures, and who were unaware of their HIV serostatus or were living with HIV but not engaged in HIV treatment, were eligible to participate.

### Community Engagement and Education

To enhance community engagement and for following principles of Good Participatory Practice to ensure ethical and scientific integrity [25,26], a Task Force composed of TW community representatives was convened for the study. The Task Force, acting as a community advisory board, informed the investigators on community issues, advised on study design, supported development of educational programs and campaigns, and facilitated collaborations with the study population. The Task Force also played a pivotal role in creating the *Féminas* house, as a separate stand-alone community facility and space for health care, support, and community programming placed at Epicentro. Community mobilization activities conducted at this setting were coordinated by a member of the TW community. Tailored annual community engagement and education plans were designed in advance of study implementation to include the following: (1) formative research for stakeholder identification and educational material validation; (2) community awareness activities; and (3) study communication, including development of educational materials, community consultations, and communication of study results.

### Training

Formative research informed the development of training plans to educate study providers, peer health navigators, and community stakeholders on the hormone therapy intervention. TW community representatives and health care providers received medical education training in health care needs, services, and strategies including a gender-affirmative approach to transgender medical care, feminizing hormone therapy, managing HIV infection, and the peer health navigation to improve linkage and retention in care.

### Recruitment

Purposive sampling was used to recruit potential participants. Peer recruiters conducted outreach work by visiting TW-specific socialization venues, including discotheques, bars, erotic movie theaters, sex work areas, beauty parlors, volleyball courts, and others. In the field, recruiters approached their peers and asked for their verbal consent to receive information about the study design and criteria for participation. Flyers containing educational material and contact information were distributed after these informative meetings to refer potential participants to the research site for screening. This recruitment strategy was complemented by social media initiatives to promote study participation.

### Screening

Counselors explained the study objectives to volunteers and obtained written informed consent to be screened for participation, HIV and STI testing, sample storage for future testing in ancillary studies, and contact for future studies. Participants underwent a computer-assisted self-interview (CASI) to assess demographics, gender identity, sexual orientation and role, sexual risk behavior, previous HIV testing and diagnosis, history of body enhancement procedures and hormone therapy use, life expectations, problems and barriers perceived because of being TW, HIV testing history, and prior engagement in care, if HIV positive. Counselors assisted participants in cases of computer unfamiliarity or literacy challenges. Physicians obtained a brief medical history and performed a targeted physical examination. A peripheral blood sample was obtained for assessment of HIV, syphilis, hepatitis B virus (HBV) and hepatitis C virus (HCV) infection, as well as hematology and biochemistry laboratory tests. Participants underwent oropharyngeal and rectal swabbing and provided a urine sample for the diagnosis of *Neisseria gonorrhoeae* and *Chlamydia trachomatis* infection. An anal cytobrush was used to assess the presence of cytological abnormalities induced by human papillomavirus infection. Participants were asked to provide 2 sputum samples and undergo a chest x-ray to rule out active tuberculosis. Volunteers were convened to return to the research site in 2 weeks for the provision of results and enrollment if they were eligible and agreed to participate (Table 1).

**Table 1:** Study schedule of events.

Procedures		Screening	Postentry evaluations (months)^a^												
			0	1	2	3	4	5	6	7	8	9	10	11	12
Informed consent		X^b^	X	—^c^	—	—	—	—	—	—	—	—	—	—	—
Medical history		X	—	—	—	—	—	—	—	—	—	—	—	—	—
Medication history		X	—	—	—	—	—	—	—	—	—	—	—	—	—
Clinical assessment		X	—	X	—	X	—	—	X	—	—	X	—	—	X
Complete physical exam		X	—	—	—	—	—	—	—	—	—	—	—	—	—
Targeted physical exam			—	X	—	X	—	—	X	—	—	X	—	—	X
Computer-assisted self-interview		X	—	—	—	X	—	—	X		—	X	—	—	X
Complete blood count		X	—	X	—	X	—	—	X	—	—	—	—	—	X
Liver and renal function tests		X	—	X	—	X	—	—	X	—	—	—	—	—	X
Fasting glucose and lipids			X	—	—	—	—	—	X	—	—	—	—	—	X
Estradiol and total testosterone			X	—	—	X	—	—	X	—	—	—	—	—	X
Chest x ray and sputum samples		X	—	—	—	—	—	—	—	—	—	—	—	—	—
Risk reduction counseling, condoms, and lubricants		X	X	—	—	X	—	—	X	—	—	X	—	—	X
Sampling for *Neisseria gonorrhoeae* and *Chlamydia trachomatis* testing		X	—	—	—	—	—	—	—	—	—	—	—	—	—
Sampling for anal cytological abnormalities		X	—	—	—	—	—	—	—	—	—	—	—	—	—
Syphilis serology		X	—	—	—	X	—	—	X	—	—	X	—	—	X
Hepatitis B and hepatitis C serology		X	—	—	—	—	—	—	—	—	—	—	—	—	—
HIV serology		X	—	—	—	—	—	—	—	—	—	—	—	—	—
**Care of HIV negative**			—	—	—	—	—	—	—	—	—	—	—	—	—
	HIV serology		—	—	—	X	—	—	X	—	—	X	—	—	X
	Pre-exposure prophylaxis dispensation		X	X	—	X	—	—	X	—	—	X	—	—	X
**Care of HIV positive**			—	—	—	—	—	—	—	—	—	—	—	—	—
	CD4+ cell count		X	—	—	—	—	—	X	—	—	—	—	—	X
	HIV-1 RNA		X	—	—	X	—	—	X	—	—	—	—	—	X
	Antiretroviral treatment dispensation		X	X	—	X	—	—	X	—	—	X	—	—	X
**Feminizing hormone therapy**			—	—	—	—	—	—	—	—	—	—	—	—	—
	Adherence counseling and education		X	X	—	X	—	—	X	—	—	X	—	—	X
	Dispensation		X	X	X	X	X	X	X	X	X	X	X	X	X
Peer health navigation support		X	X	X	X	X	X	X	X	X	X	X	X	X	X

^a^Visits to occur every 30 days ± 5 days.

^b^The procedure is indicated at the specific study visit.

^c^Not applicable.

### Enrollment

Peer health navigators remained in contact with screened participants and facilitated their return to the research site for enrollment. At the site, a physician assessed eligibility, which included TW who resided in Lima and had normal hematology and biochemistry laboratory results. Individuals presenting with active tuberculosis; history of pancreatitis; severe or depending alcohol or drug consumption; severe medical comorbidity; reporting the use of immunosuppressive, nephrotoxic, or hepatotoxic therapy; or having any other health condition that in the opinion of the investigator would interfere with the evaluation of the study objectives were excluded. Volunteers received a detailed explanation of the risks and benefits of the medical intervention. Consenting participants underwent a CASI questionnaire, which included questions about personal and social network support for resilience to societal stigma and discrimination, hormone therapy expectations, and housing. A peripheral blood sample was obtained for assessment of baseline fasting glucose and lipids, estradiol, and total testosterone levels. All participants were prescribed and dispensed feminizing hormone therapy and invited to initiate HIV prevention and care as described in Table 1.

### Follow-Up

Participants were asked to return to research site for study visits at months 1, 3, 6, 9, and 12, which included clinical and laboratory safety assessment, and hormone therapy adherence counseling and education following standard protocols [27]. At quarterly visits, participants underwent HIV (for those negatives) and syphilis testing, and answered a CASI questionnaire on gender identity and body image sexual risk behavior, substance use, attitudes toward HIV testing, personal and social network support, hormone therapy expectations, mental health, adherence to hormone therapy, and involvement in TW community building activities (Table 1). The occurrence of laboratory grade ≥2 and clinical grade ≥3 adverse events were monitored in all follow-up visits. All participants were instructed to report the occurrence of unintended effects of the study at any time. Peer health navigation facilitated retention in care and promoted adherence to study procedures, HIV prevention and care, and hormone therapy by visiting participants at home, work or socialization venues, or by contacting them by social media and phone.

### Regimen for Feminizing Hormone Therapy

Hormone therapy followed Fenway Health's protocol providing medical care of transgender persons [27]. Hormones were dispensed on site on a monthly basis. Follow-up dosages were individualized in response to clinical efficacy or the occurrence of adverse events. Estradiol valerate was initiated at 2 mg PO daily and increased to 4 mg after 4 to 12 weeks. Antiandrogen therapy with spironolactone started at a dose of 50 mg daily and indicated to be increased every 4 weeks to 200 mg daily.

### HIV and Sexually Transmitted Infection Prevention and Care

All participants received risk reduction counseling, condoms, and lubricants when tested for HIV and/or STI. Participants diagnosed with HIV infection were invited to be linked to HIV care at IMPACTA, placed 4 blocks away from the research site Epicentro, where local standard care was offered, including initiation of antiretroviral therapy (ART) and assessment of baseline and quarterly plasma HIV-1 RNA and CD4+ lymphocyte cell count monitoring [28]. HIV-negative participants were invited to initiate standard HIV pre-exposure prophylaxis (PrEP) with daily oral emtricitabine/tenofovir disoproxil fumarate [29], which was available at the research site at no cost. Participants diagnosed with STI received international standard treatment and those susceptible to HBV were referred for vaccination [30].

### Peer Health Navigation

Socially well-connected community members representing the diverse subcultures of TW in the city and with preexisting knowledge of local HIV and related social resources were convened and provided with skills and confidence to help TW self-manage their health care needs with the ultimate goal to facilitate engagement in study procedures and retention in follow-up. Training in peer navigation followed Fenway Health's protocol for HIV system navigation [18]. Navigators were trained in several widely used frameworks, including the strengths-based perspective from social work practice, motivational interviewing, and stages of change. The strengths-based approach identifies, supports, and builds on patients’ individual capacities, competencies, values, and hopes toward the goal of personal empowerment and resilience. Motivational interviewing is a theory-based, empirically valid approach that helps to change behaviors by examining and overcoming ambivalence that keeps many people from changing. Similar to motivational interviewing, the transtheoretical (*stages of change*) model provides a way to assess patients’ readiness to adopt health-promoting behaviors. In addition to the initial 3-day training workshop, navigators received 4-hour monthly re-training sessions, in which barriers and facilitators experienced by navigating the system were shared and discussed [18].

### Laboratory Procedures

HIV antibody screening was conducted on whole blood using Determine HIV-1/2 (Alere, Waltham, MA), a point-of-care third-generation HIV antibody test. Positive samples were sent for confirmatory testing by means of indirect immunofluorescence to the Peru National Institute of Health (NIH), unless a previous positive confirmatory result was identified in the Peru NIH database. Plasma HIV-1 RNA and CD4+ T-cell count assessments were also conducted at the Peru NIH. Antibodies to *Treponema pallidum* were detected using a rapid plasma reagin test (RPR Quicktest, Stanbio, Boerne, TX) and confirmed by a *T. pallidum* particle agglutination assay (DR0530, TPPA Test, Oxoid Limited, Basingstoke, UK). Both HBV (HBsAg, anti-HBc, and anti-HBs) and anti-HCV serologies were tested by means of chemoluminescent microparticle immunoassay (Architect, Abbott, Abbott Park, IL). *N. gonorrhoeae* and *C. trachomatis* infections were diagnosed by GeneXpert CT/NG (Cepheid, Sunnyvale, CA). Cytological slides were manually colored using standard Papanicolau staining. Sputum testing for tuberculosis used the Ziehl-Neelsen stain. Hematology (XS-1000i Sysmex America, Inc, Lincolnshire, IL), biochemistry (IFCC Vitros, Ortho Clinical Diagnostics, Raritan, NJ), and hormone (Cobas E-601, Roche Diagnostics, Indianapolis, IN) tests were assessed automatically. Unless previously specified, all tests other than hepatitis serology and hormone tests (at Anglolab laboratory) were conducted at the IMPACTA PERU Clinical Trials Unit Laboratory. All test results were provided to participants within a period of 14 days.

### Endpoints

Endpoints at baseline were as follows: (1) proportion of participants who had ever been tested for HIV and (2) proportion of participants newly diagnosed as HIV positive. At the end of the intervention (12-month follow-up), the endpoints were (1) proportion of HIV-negative participants at baseline who were motivated, accessed, and effectively underwent quarterly HIV testing, used condoms or PrEP [20] and (2) proportion of HIV-positive participants at baseline who linked to and were retained in care, adhered to ART, and resulted in viral suppression [19]. Occurrence of study endpoints were assessed by an endpoint adjudication committee composed by the protocol team, led by the 3 study principal investigators.

### Data Management and Monitoring

All participants’ data were anonymized but not deidentified. All participants were identified with a unique alphanumeric code. The link between the participants’ codes and identification was restricted to the study coordinator. It was stored in an electronic file and double password protected. Data were monitored for completeness, consistency, and accuracy, as well as for the occurrence of adverse events, in a biweekly basis by a protocol team led by the 3 study principal investigators.

### Statistical Considerations

The target enrollment was 220 TW for a total of 200 completers (>90% retention during 12-months of follow-up) (Table 2). On the basis of these estimated percentages and assuming a moderate correlation across measurements (ρ=.30) [31], the power to detect statistically significant differences across follow-up for all HIV cascade outcomes is greater than 0.80. Quantitative analyses will be implemented in Stata SE 13.0 (Stata Corp, College Station, TX) software using 2-tailed tests of significance, with statistical significance at the alpha .05 level. A generalized estimating equation (GEE) approach will be used [31-33] with robust standard errors, which is an extension of regression analysis that properly accounts for repeated measures. Descriptive statistics will be obtained to summarize all variables. Bivariate tests (*t* tests/chi-square adjusted longitudinally using GEE) will examine changes in the proportion of TW across outcomes of interest, followed by multivariable longitudinal models adjusted for covariates (eg, age). GEE models use the number of observations as data points over time (5 visits×200 TW=1000 observations). Multiple imputation [34,35] will be used assuming missingness is completely random.

**Table 2:** Projected sample sizes across HIV prevention and treatment cascade outcomes (N=200).

Cascade stage		Baseline, n (%)	12-months, n (%)	Difference in proportions	Outcomes
HIV test and risk reduction counseling in past 3 months		75 (37.5)	195 (95.0)	57.5	Increase in the proportion of TW who know their HIV status
**HIV positive**		46 (23.0)	66 (33.0)	10.0	Identify new HIV infections in TW
	Linked to care	22 (47.8)	66 (100.0)	52.2	Increase in the proportion of HIV-positive TW linked to care
	Retained in care	9 (40.9)	60 (90.9)	50.0	Increase in the proportion of HIV-positive TW retained in care and treatment
	Adhered to ART^a^	11 (50.0)	60 (90.9)	40.9	Increase in the proportion of HIV-positive TW prescribed ART
	Viral suppressed	5 (45.5)	50 (83.3)	37.8	Increase in the proportion of HIV positive TW who are virally suppressed
**HIV negative**		154 (77.0)	134 (67.0)	10.0	Identify high-risk HIV-uninfected TW
	Completed referrals to HIV prevention services	35 (22.7)	70 (52.2)	29.5	Link high-risk HIV-uninfected TW to HIV prevention services, including access to pre-exposure prophylaxis
STI^b^ screening, risk reduction counseling, and treatment in past 3 months		50 (25.0)	200 (100.0)	75.0	Increase in the proportion of TW screened and treated for STIs

^a^ART: antiretroviral therapy.

^b^STI: sexually transmitted infection.

Secondary analyses characterizing patterns of HIV virologic suppression over time will also be implemented. HIV cascade outcomes are also time to event data beyond the achievement of the dichotomous outcomes we propose. Therefore, data that allow us to capture the time at which an event of particular interest occurred (ie, virological suppression) will be collected. Patterns of change in biomarkers (ie, CD4+ cell count decrease) or the association between the primary endpoint and features of the longitudinal profiles are of interest. We will conduct joint modeling that enables longitudinal repeated biomarker measurements and survival processes to be modeled simultaneously while taking into account the association between them [36-38]. The random effects model for longitudinal data will be included in the survival model. Joint modeling provides less biased estimates and more efficient inferences than a 2-stage modeling approach [39-41]. We will use SAS 9.4 (SAS Institute Inc, Cary, NC) [42]. The joint modeling approach is well suited for our study because there is no control group (which is best suited for implementation science), and within-person change across time is being assessed.

### Protection of Human Subjects

All participants were reimbursed approximately US $13 in local currency (40 Soles) at each study visit. The study protocol and its amendments, informed consent forms, and recruitment and educational materials were approved by the IMPACTA's Institutional Bioethics Committee in compliance with all applicable Peruvian and US Federal regulations governing the protection of human subjects. All participants provided written informed consent for study screening and participation, receiving feminizing hormone therapy, HIV and STI testing, sample storage for future testing, and contact for future studies. The study protocol is registered at Peru National Institute of Health (Registro Nacional de Investigaciones en Salud EI00000345; dated on May 29, 2018) and Clinicaltrials.gov (Registration Number: NCT03757117; dated on November 28, 2018).

### Poststudy Care Access

At the end of the intervention, all participants will be invited to continue receiving HIV and STI prevention and treatment at IMPACTA or referred to health care centers of the Peru Ministry of Health to receive it. It is expected that by time the study ends, feminizing hormone therapy will become available at public health care centers as part of the Ministry of Health policies to provide integrated HIV prevention and care to TW [43].

### Dissemination

When intervention is near completion, the study team will prepare communication plans for disseminating and interpreting study results. We will host a preparatory activity of study results as a forum on the implications of the intervention targeting Ministry of Health representatives and the scientific and community stakeholders. In this forum, we will present a draft version *Best Practices* for dissemination of integrated TW health care services and ask audience for input that will be further incorporated. Thereafter, we will prepare the TW community for the disclosure of the results in a participant appreciation event.

## Results

Patient recruitment started in October 2016 and final inclusion was March 2017. The cohort ended follow-up in March 2018. Data analysis is currently underway, and the first results are expected to be submitted for publication in June 2019.

## Discussion

The *Féminas* study will investigate the feasibility and acceptability of an innovative service delivery model that integrates HIV prevention and treatment services with gender-affirming transgender medical care supported by peer navigation to improve the HIV prevention and care cascades among TW in Lima, Peru. To our knowledge, interventional research has not yet combined these 3 aspects to assess HIV outcomes across 12-months of prospective follow-up for TW primary medical care. The competent provision of feminizing hormones along with colocalized services targeting the HIV prevention and treatment cascades, supported by peer navigation, appears to be an attractive model for TW and an important aspect for future implementation of these services to address the HIV epidemic for TW.

For the success of the intervention, transgender community engagement is essential [17,23]. We promoted community mobilization through the implementation of community engagement and education plans, which were structured by members of the TW community in response to their health needs. Study procedures were implemented at a culturally competent CBO accustomed to regularly providing services for TW residents in Lima. The TW Task Force was particularly crucial in the beginning of the project to gather input on the study design and implementation and offer a vision of the model that would be maximally responsive to the needs of the population.

The coordinator of the *Féminas* house had a challenging role, given her proximity to the community and the many demands placed upon her in terms of the project and by her peers. It is vital that the TW community is represented in the staff, particularly in a leading role such as coordinator [44]. At the same time, caution is warranted so as not to tokenize or overburden this individual in her professional work as a transgender peer. The coordinator position must have supportive and ongoing supervision that is gender affirming with a person (or people) knowledgeable about TW communities and not necessarily within trans communities, preferably clinical supervision. This supervision needs to include ongoing assistance troubleshooting any number of challenging situations that can arise between the personal and professional aspects of project coordination and strategizing how best to manage *insider* trans women community politics with other peer leaders and organizations.

Peer navigation is vital for the *Féminas* model of care [18]. At every initial and subsequent point of contact and visit, participants have social, medical, legal, and other needs, which necessitate referrals and assistance. The peer health navigation takes a great deal of staff time and effort, much more than originally anticipated and budgeted given the many challenges and barriers to services facing TW in Lima in the broader context of social exclusion and economic marginalization. In HIV service models, peer health navigators generally have a caseload of 20 to 40 HIV-positive individuals, depending on the number of complex needs individuals they are supporting and availability and quality of health care services and systems [45,46]. For the *Féminas* model, a maximum of 25 TW peers per navigator would be ideal. Appropriate and realistic staffing and effort for peer health navigation is an important future consideration for the model. In addition, there are many types of and approaches to peer navigation; however, surprisingly few curricula exist to operationalize and skill-build TW peer health navigators. Compiling and disseminating a manualized curriculum to train on peer health navigation represents an important next step.

Our study has several limitations. First, as a pilot single-arm study without a standard-of-care arm, this interventional research cannot assess the effectiveness of the intervention. Nevertheless, this study will play a key role in the development or refinement of a potential future intervention that we anticipate could be tested for efficacy in a later trial [47]. Second, purposive sampling was used to convene potential participants to the study; therefore, study participants do not necessarily represent the TW community in Lima. Sampling may have been biased by the peer recruiters’ knowledge of, and access to, different subgroups of TW. In addition, HIV-positive TW already in care were not included in the study. Thus, we will not be able to assess whether an integrated model of gender-affirming hormones and HIV care can improve HIV cascade outcomes for TW living with HIV and already receiving care.

The anticipated challenges inherent in studying at-risk populations include high participant attrition and low enrollment, as well as high rates of loss to follow-up. We have built a system robust against attrition through comprehensive collection of information on how to locate and contact participants, active tracking and engagement of participants between appointments using peer navigation, and reimbursement to minimize loss to follow-up. The study will gather information about retention efforts for TW in Lima, including diverse strategies to minimize attrition. It is anticipated that integration and colocalization of hormones and HIV prevention and care will support TW retention and facilitate engagement in the study [48].

Findings from this implementation science study will inform future policies and research, including evaluation of the efficacy of the *Féminas* intervention in a future randomized controlled trial.

## Abbreviations

ART: antiretroviral therapy
CASI: computer-assisted self-interview
CBO: community-based organization
GEE: generalized estimating equation
HBV: hepatitis B virus
HCV: hepatitis C virus
IMPACTA: Asociacion Civil Impacta Salud y Educacion
NIH: National Institute of Health
PrEP: pre-exposure prophylaxis
STI: sexually transmitted infection
TW: transgender women

## Appendix

Multimedia Appendix 1 Study protocol funder's original peer-review reports.
