# Supplementary material for: Integration of Gender-Affirming Primary Care and Peer Navigation With HIV Prevention and Treatment Services to Improve the Health of Transgender Women: Protocol for a Prospective Longitudinal Cohort Study
Source: JMIR Res Protoc. 2019 Jun 27;8(6):e14091. doi: 10.2196/14091 (PMC6620883; doi:10.2196/14091)
Supplement: Multimedia Appendix 1 [file resprot_v8i6e14091_app1.pdf]

Javier Lama, M.D., M.P.H.
Asociacion Civil Impacta Saud y Educacion, Lima, Peru
$876,684 (#109071)
Gender-Affirmative Transgender Care to Improve the HIV Treatment Cascade

Overview of review discussion

The reviewers were enthusiastic about this proposal, for its responsiveness to and focus on TW needs. Overall, the proposal is very strong, but some details remain to be clarified and/or determined.

The delivery model is not clearly explained. Concerns were raised about exclusion of known positive TW who have fallen out of care, and potential exclusion of TW not seeking hormone therapy. The location of activities was also not clear. Are participants supposed to go to EPICENTRO for their hormones and other trans-related care, and then go to IMPACTA for HIV care? It would be good to make this truly seamless care. If all is being provided at EPICENTRO, what is the funding for IMPACTA staff for?

Sustainability was another major concern. It isn't clear from the proposal if and how hormone treatment is usually covered. Where will TW get their care and hormones after study participation is complete? Availability of PrEP was also unclear. Although Truvada is finally licensed in Peru, PrEP is not part of a national program. Is there a plan for delivery of PrEP after study completion?

Some of the study procedures were also unclear and seemed inconsistent. Will HIV positives get STI screening? Will HIV negatives also receive hormone therapy? Are the lab costs covered under usual care?

Reviewers remained enthusiastic, but felt these were important issues to address.

Reviewer 1

<u>Relevance/Potential Impact</u>

The potential impact is high, given the HIV disparities among the TW population and lack of access to safe TW-related medical care.

<u>Innovative Application of Proven Intervention(s)</u>

The proposal aims to extend a well-tested model of TW care developed by the Fenway co-investigators to TW in Peru, via a previously successful collaboration between IMPACTA and Fenway. The 3-step approach of formative research, prospective study, capped by a cost-effectiveness analysis feels “complete”, innovative, and will bolster eventual expansion.

The sustainability of the intervention is not addressed in two key ways. First, the delivery system (and study design) of Figure 1 ends at 12 months, but the investigators do not address how the ongoing hormonal, HIV/STI prevention, or HIV care-related needs might continue to be met after study participation ends. Second, although the economic viability of the intervention will be

nicely quantified in Phase III, there is no detail provided on how this might translate into action that might support the continuation of the intervention. It is also unclear what the long-term implementation vision for the intervention would be. From the provider focus group description and training component, it seems that the hope is that gender-affirming care would eventually be implemented in government-run HIV care facilities, but the intervention is being tested in a CBO. Because the authors describe a landscape of essentially zero access to gender-affirming care, perhaps a few well-equipped CBOs is the optimal model for service delivery that they envision?

Approach
The investigators have proposed an approach that merges an established local HIV research group (IMPACTA), TW-affirming CBO (EPICENTRO), and US-based academic/implementation partner (Fenway). The approach is generally thoughtful and the investigators have adequately addressed many of the statistical concerns originally noted. However, there remains important lack of clarity about the study design and the outcomes:

Formative research (part B): Nice and rich methodology described. TW not living with an HIV diagnosis are being sought, but this is at odds with the content of the focus groups including “engagement in HIV primary care”. It seems that including those diagnosed would be needed.

Population (part D): No rationale is provided for excluding people living with an HIV diagnosis, who are a large portion of the population and would benefit from the care continuum interventions and gender-affirming care, per Figure 1. Relatedly, some data on what proportion of those who are HIV-infected are diagnosed vs. undiagnosed are needed to understand the importance of this criterion and to substantiate some of the numbers in Table 2’s sample size projections. A major source of confusion in Table 2 is there being non-zero HIV care continuum outcomes at baseline, if the study is to include only newly diagnosed individuals.

Recruitment (part E): The investigators addressed the request from the funder to justify the sampling procedure, by referring to successful usage for sentinel surveillance surveys. However, any details of what exactly will occur, besides ‘outreach’ via socially connected TW and staff in venues, is omitted.

Intervention (part F): The description of the intervention lacks a clear road map for the study visits. The screening visit and subsequent visit schedule are not defined outside of Table 1. It seems that visits will be split in two locations, with HIV-related activities occurring at IMPACTA and TW hormone therapy occurring at EPICENTRO. Because the hormone therapy is a required procedure (see next paragraph), perhaps EPICENTRO is the primary site of the study visits, but this is unclear and only inferred from Table 1. With HIV endpoints only (testing, linkage, suppression), is their achievement mainly a function of whether the participants walk from EPICENTRO to IMPACTA after their gender affirming-care visits, possibly with EPICENTRO staff because they are listed as the HIV care providers in the budget justification? The endpoints should be better-defined, with some secondary endpoints that assess success of the gender-affirming care.

As an aside, given this description, it appears that all participants must elect for hormone therapy to participate, but this isn’t part of the official inclusion criteria and reads as an extreme

and possibly unethical requirement for access to an HIV intervention. Thus it seems that TW who opt out of the hormone procedures cannot participate in the study and receive the other supportive TW-related and HIV services, even though all study endpoints are HIV-related.

Cost-saving analysis: This is an exciting component to the project, although perhaps as proposed, it is more complex than need be. The statement that the "intervention is deemed cost-saving if it costs less than the discounted treatment cost per infection averted" seems strange because this implies that each person reached by the intervention prevents 1 new HIV infection (NNT=1), which is overly optimistic.

Investigator Qualifications
This is a well-qualified team, both in Peru and the US. No deficiencies here.

Institutional Commitment
All assurances included, with no concerns.

Community Engagement
The inclusion of EPICENTRO as the site of providing the gender-affirming care, but as detailed above, this appears to split the study visits across two locations and has unclear implications for scale-up. EPICENTRO personnel are included as key personnel. Substantial and meaningful community engagement will occur during the formative research phase.

Administrative Concerns
The approach for Phase 2 indicates that the HIV care will be provided at IMPACTA, but the IMPACTA staff in the budget are providing only study coordination. EPICENTRO staff are listed as providing HIV care, presumably at IMPACTA. Not a major concern, but confusing.

Reviewer 2
Relevance/Potential Impact

- There is a profound lack of well-described models for providing coordinated prevention services to transgender women.
- Aligned with RFP's intent.
- This model would make an important difference for TW.

Innovative Application of Proven Intervention(s)

- This is an innovative proposal and addresses substantial need.

Approach
Strengths:

- Power calculations are provided and suggest reasonable power against a combined initiation and retention endpoint.
- Use of peer health interventions is a strength.
- Evidence of ability to reach TW.
- Mixed methods approach is useful.
- Research plan is well described.
- Likely to be successful based on previous work by the study team.

Weaknesses:

- The status of Truvada for HIV prevention is not explicitly described. It is stated that is licensed and available to purchase, but it is not stated whether additional approvals are needed to use Truvada for prophylaxis. It is possible that Dr. Clark's project will have addressed this issue.
- The power calculations for part 2 propose high power to detect significant differences across follow-up for all cascade outcomes. Some of the changes in proportions proposed are quite large (e.g., 75% increase in proportion of STI screening). Observed changes are unlikely to be this large.

Investigator Qualifications

- Strong investigator team
- Great partnership between IMPACTA and Fenway
- Dr. Lama is a strong leader for the project
- Collaboration by Dr. Mayer provides an important link to existing Fenway programs of integrated care
- Sari Reisner brings critical expertise in service provision for Trans people.

Institutional Commitment

- A letter of institutional commitment is provided

Community Engagement

- There are no letters of support from community partners except for EpiCentro, which is a funded research partner. Other letters of support are from academic partners.
- Formation of a TW Task for is proposed but there is not a clear indication of capacity to do this.

Administrative Concerns

- None
